# The Greater Houston Area Bipolar Registry—Clinical and Neurobiological Trajectories of Children and Adolescents With Bipolar Disorders and High-Risk Unaffected Offspring

**DOI:** 10.3389/fpsyt.2021.671840

**Published:** 2021-06-04

**Authors:** Alexandre Paim Diaz, Valeria A. Cuellar, Elizabeth L. Vinson, Robert Suchting, Kathryn Durkin, Brisa S. Fernandes, Giselli Scaini, Iram Kazimi, Giovana B. Zunta-Soares, João Quevedo, Marsal Sanches, Jair C. Soares

**Affiliations:** ^1^Center of Excellence on Mood Disorders, McGovern Medical School, Faillace Department of Psychiatry and Behavioral Sciences, The University of Texas Health Science Center at Houston, Houston, TX, United States; ^2^Translational Psychiatry Program, Faillace Department of Psychiatry and Behavioral Sciences, McGovern Medical School, The University of Texas Health Science Center at Houston, Houston, TX, United States; ^3^Methodist Hospital, Houston, TX, United States; ^4^Neuroscience Graduate Program, The University of Texas MD Anderson Cancer Center UTHealth Graduate School of Biomedical Sciences, Houston, TX, United States; ^5^Translational Psychiatry Laboratory, Graduate Program in Health Sciences, University of Southern Santa Catarina, Criciúma, SC, Brazil

**Keywords:** adolescent, bipolar disorders, child, longitudinal studies, neurobiology

## Abstract

The aims of this article are to discuss the rationale, design, and procedures of the Greater Houston Area Bipolar Registry (HBR), which aims at contributing to the effort involved in the investigation of neurobiological mechanisms underlying bipolar disorder (BD) as well as to identify clinical and neurobiological markers able to predict BD clinical course. The article will also briefly discuss examples of other initiatives that have made fundamental contributions to the field. This will be a longitudinal study with participants aged 6–17 at the time of enrollment. Participants will be required to meet diagnostic criteria for BD, or to be offspring of a parent with BD. We will also enroll healthy controls. Besides clinical information, which includes neurocognitive performance, participants will be asked to provide blood and saliva samples as well as to perform neuroimaging exams at baseline and follow-ups. Several studies point to the existence of genetic, inflammatory, and brain imaging alterations between individuals at higher genetic risk for BD compared with healthy controls. Longitudinal designs have shown high conversion rates to BD among high-risk offspring, with attempts to identify clinical predictors of disease onset, as well as clarifying the burden associated with environmental stressors. The HBR will help in the worldwide effort investigating the clinical course and neurobiological mechanisms of affected and high-risk children and adolescents with BD.

## Introduction

According to the National Comorbidity Study-Adolescent Supplement (NCS-A), the lifetime prevalence of Bipolar disorder (BD) I or II among adolescents is 2.9% (1), with 30% of adults with BD presenting with an age of onset of the disease before 17 years old (2, 3). Early-onset BD, defined as onset before age 18, is associated with several poor outcomes, including higher rates of lifetime comorbidity with other psychiatric disorders, delayed treatment-seeking, and a higher likelihood of suicide attempts (3–6), as well as a higher caregiver's burden and costs (7, 8).

Scientific efforts have focused on advancing knowledge about BD in children and adolescents in order to prevent or minimize its significant individual, familial, and social impact. For instance, the investigation of variables associated with distinct outcomes in children and adolescents with BD may allow the identification of features indicating the need for specific interventions. Duffy et al. (9) found that a history of hallucinations, delusions of guilt, and many different psychotic symptoms were associated with suicidal ideation in young patients with BD type I (9). Suicide risk in children and adolescents with BD was also associated with sleep disorders in the study of Stanley et al. (10). Cazala et al. (11) investigated the association between childhood trauma with irritability and aggressive and suicidal behaviors in pediatric participants with BD across sex. The authors found that a history of physical abuse was associated with suicidal thoughts in females. In contrast, a history of emotional abuse was associated with greater violence against property in males (11).

Likewise, the study of high-risk individuals may help in the clarification of the neurobiological mechanisms underlying BD and in the identification of potential biomarkers for diagnosis, disease vulnerability and risk, and neuroprogression (12, 13). In particular, the identification of risk or disease vulnerability markers could help in the identification of strategies for early intervention to implement primary prevention measures and improve disease course (14). A cohort study showed a 24.5% cumulative incidence of BD spectrum disorders in a sample of unaffected offspring of parents with BD over a mean follow-up period of 8 years (15), which is much higher than the incidence in the general population (16, 17). These young individuals also present with increased risk of a non-BD affective disorder, anxiety, substance use disorders, and cognitive impairment; the last is also a hallmark of BD that is also found in offspring of parents with BD (18–20).

Fries et al. (21) reported different patterns of DNA methylation across groups of children and adolescents with BD and unaffected offspring (21). The authors found distinct expression levels of the glucocorticoid receptor (GR) signaling pathway genes in patients with BD, and also in high-risk participants, as compared to controls, which may reflect the reported association between stress and the pathophysiology of BD (22, 23). Different profiles of immune growth factors in unaffected offspring of a parent with BD and healthy controls were also found in adolescents (24). Negative correlations between duration of BD and hippocampal volumes, as well as higher brain levels of inflammatory and innate immune markers in young individuals with BD who died by suicide, suggest that disease neuroprogression is already present in this population (25, 26). Coupled with information about life stress events, neurodevelopmental, and environmental characteristics, these studies may be able to explore potential moderators and mediators of different outcomes (27, 28).

Moreover, longitudinal epidemiological studies of high-risk individuals allow the search for biomarkers potentially able to predict specific clinical outcomes and disease stage in bipolar disorder (29–31), as well as the clarification of different trajectories of the disorder (32). For instance, a longitudinal study with 135 young participants with BD (mean age = 16 years old) has identified distinct patterns of cognitive functioning in the sample. The results showed that those children and adolescents in the “persistently low” cognitive performance group presented poorer course not only regarding their mood but also concerning their academic and social functioning (32). Unaffected siblings of BD probands may present a similar cognitive function pattern compared to BD individuals, with verbal memory as a potential endophenotype considering the worse performance of unaffected siblings than healthy controls reported by Russo et al. (33). A meta-analysis that included 14 studies with first-degree BD relatives found a significantly worse cognitive performance of these individuals than healthy controls in all domains investigated, despite small effect sizes (34). However, in a cross-sectional study of our group with a machine learning algorithm, cognition measures did not differentiate between unaffected offspring and healthy controls in a pediatric sample (35). Addressing children and adolescents with mood disorders as well as healthy controls through dimensional clinical measures and several levels of analysis may help identify more homogeneous clusters of individuals with better clinical predictability (36, 37).

The Greater Houston Area Bipolar Registry (HBR), conceptualized and implemented at the UTHealth Center of Excellence on Mood Disorders, is one of the current initiatives aiming at providing a detailed clinical and neurobiological longitudinal evaluation of children and adolescents with BD and high-risk unaffected offspring. In this paper, we will discuss its design, procedures, and some examples of other initiatives that have made significant contributions to the field.

## Methods and Analysis

### Subjects

Subjects will be either male or female, aged 6–17, at the time of enrollment. Participants will be required to meet Diagnostic and Statistical Manual of Mental Disorders, fifth edition (DSM-5) diagnostic criteria for BD types I, II, or other specified bipolar and related disorder, or to be offspring of a parent with BD (types I or II) confirmed by a clinical interview [MINI International Neuropsychiatric Interview for children and adolescents English version 7.0.2 for DSM-5 (M.I.N.I. KID)] (38). We will also enroll subjects without personal or family history of psychiatric disorders as healthy control subjects for comparison. All participants with a history of autism spectrum disorder, intellectual disability, a severe neurological disorder that affects cognitive status (e.g., epilepsy, traumatic brain injury, tubular sclerosis), schizophrenia, and uncontrolled or severe medical problem will be excluded. Healthy controls with any family history of Bipolar Disorder, Psychotic Disorder, Schizophrenia, or Schizoaffective disorder in a first-degree relative and any family history of a neurological condition in a first-degree relative will also be excluded from participation in the control group. Although we will use a clinical interview to rule out psychiatric diagnosis in healthy controls' parents, neurological conditions and psychiatric conditions of other first-degree relatives will be collected according with parent/legal guardian information, which can limit the accuracy of the data.

These subjects will be recruited from the Harris County Psychiatric Center (HCPC), University of Texas (UT) Health Science Center at Houston Child and Adolescent Outpatient Psychiatry Clinic, referrals from other health providers, as well as self-referrals. We will be advertising the study through the UTHealth Center of Excellence on Mood Disorders website, UTHealth Media Relations, radio/television ads, public transportation advertisement sections, and flyers/brochures. The recruitment from different sources will help to keep the samples as diverse and representative of the metropolitan area of Houston as possible.

### Procedures

#### Screening Visit

A member of the research team will explain the study to potential participants and their parents/legal guardians. Concerns or questions will be addressed at that time. Subjects who agree to participate, as well as a parent or legal representative, will be asked to sign a consent form. Each assessment will be conducted in English; thus, the participants will have to be fluent in English but not necessarily English native-speakers; this was decided in order to increase generalizability. Research assessments will be administered to the child/adolescent and, preferably, to both parents. In case both parents are not available, it will be acceptable to administer the assessments to just one parent, as long as the non-assessed parent has no history of psychiatric disorder as informed by the available parent.

Each child/adolescent and parent/legal guardian will receive a battery of assessments during their in-person visit, which includes a clinical interview and psychometric scales for measuring clinical symptomatology. All assessments will be administered in a standard manner involving researcher-administered and self-report questionnaires. We will use the M.I.N.I. for DSM-5 to assess the psychiatric diagnosis of participant's parents from the three groups, children and adolescents with BD, unaffected offspring, and healthy controls (39).

#### Second Visit

Children/adolescents will qualify for the registry if they belong to one of the following three groups: (1) BD I, II, or other specified bipolar and related disorder; (2) offspring of at least one parent with BD (whose diagnosis was confirmed through a clinical interview); (3) or healthy controls. Enrollment will be monitored by a board-certified psychiatrist, who will review the diagnosis obtained through the standardized interviews performed at screening. If patients meet the criteria for study participation, they will be asked to complete a second visit. At this visit, the participant will complete self-report scales and undergo a comprehensive neuropsychological test, The Cambridge Neuropsychological Test Automated Battery-CANTAB, to assess his or her cognitive functioning (40). The CANTAB is a well-known cognitive battery and can be feasibly applied to children and adolescents with comparable task performance between native and non-native English speakers (41). In addition, the battery presents high internal consistency coefficients in children and can be administered without modification in children and adolescents, with the same tests applied to adults (41). This battery includes reaction time, motor screening task, paired associates learning, rapid visual information processing, spatial span, spatial recognition memory, match to sample visual search, stockings of Cambridge, big circle little circle, intra-extra dimensional set shift, affective go/no-go, and Cambridge gambling task. As part of our protocol, in addition to clinical and neuropsychological data, children and adolescents will be asked to provide saliva and blood samples, and their parents will complete self-reports about the children/adolescent's behavior.

#### Follow-Up Visits

Participants included in the study will be contacted for an annual re-evaluation. The research team will contact the participant's family by phone. Three attempts will be made in 1 week. If unsuccessful, an email will be sent; in case of no response in another week, the research team will send a letter to the participant. If there is no answer within the 3 months following the putative date of the 1-year follow-up, the participant will be contacted only in the next year. At the re-evaluation appointments, the subject will undergo the same interview and scales they completed at baseline. Each follow-up appointment will take ~3–4 h. Children/adolescents also will undergo the neuropsychological assessment every other year at the annual follow-up visit, which takes up to 4 h. Blood samples will also be collected at their annual follow-up visits, as well as the neuroimaging data. There are no exclusion criteria after enrollment; that is, all participants will be followed regardless of their diagnosis at the time of the subsequent assessments. Medical information, including medications in use, history of hospitalizations, and suicidal behavior, will be taken at each follow-up visit. In case of retention fail, we will discuss potential barriers to continued participation in the study. We will schedule visits according to participants and relative's convenience. Other strategies for enhancing recruitment will be to intensify advertisement and communication.

### Assessment

#### Clinical Data

For children and adolescents, we will collect information about demographics and socioeconomic status (using the Hollingshead Socioeconomic Status form). Review of medical history, childhood milestones, and medication history will be done with the child/adolescent and confirmed by their parent/legal representative. History of use of psychosocial services and psychiatric hospitalization will also be reviewed at this time. The M.I.N.I. KID for DSM-5 will be applied for diagnostic evaluation (38). The psychometric evaluation will include measures of symptomatology, suicidal behavior, global functioning, clinical impression, history of trauma, and stressful life events scales. The instruments that will be applied are detailed in Table 1.

**Table 1:** The greater Houston Area Bipolar Registry - summary of the assessments.

	**Screening visit**		**Second visit**		**Annual Follow-up**	
	**Children/adolescents^a^**	**Parents^b^**	**Children/adolescents^c^**	**Parents**	**Children/adolescents^c^**	**Parents**
Sociodemographic information	X	X			X	
Psychiatric diagnostic	X	X			X	X
Psychometric evaluation^d^	X	X		X^e^	X	X
Neuropsychological evaluation^f^			X			
Saliva sample			X	X		
Blood sample			X		X	
Neuroimaging^g^					X	

a *All children and adolescents potentially eligible*.

b *Both parents preferable*.

c *Qualified for the HBR (meets criteria for BD I, II or other specified bipolar and related disorder, offspring of parents with BD and healthy controls)*.

d *Includes psychiatric symptoms, functionality, stress life events and quality of life*.

e *Self-reports about their children behavior*.

f *At every 2 years of follow-up*.

g *Scheduled after the two baseline assessments for structural MRI, magnetic resonance spectroscopy (MRS), diffusion tension imaging (DTI), and resting-state functional MRI (fMRI)*.

The M.I.N.I. for DSM-5 will be utilized to confirm the psychiatric diagnosis in parents. This scale will also be used for enrolled participants who become adults (i.e., 18 years old) in their annual follow up visit (39). Psychometric scales for assessing current mood, clinical impression, children/adolescents' symptomatology, and pubertal development, as well as family environment and stressful life events, will also be administered.

Annual follow-up visits will include all participants. The same clinical interview and psychometric measures will be applied, except neuropsychological evaluation for children/adolescents, which will be collected every 2 years. At the time of the follow-up visits, we will also collect vital signs and update the following information: General Information, Medical History, Family Psychiatric History, Medication History, and Suicide History.

#### Neurobiological Information

##### Blood and saliva samples

All subjects who qualify for the HBR study will be invited, at the time of their inclusion in the study and at each one of the yearly follow-ups, to provide one blood sample of ~34–36 ml (one lavender top vacutainer tube with K2 EDTA and three heparin tubes) at the baseline and follow-up visits for biomarker studies. If a participant refuses to provide a blood sample, he or she will still be included in the study. Immediately after the blood drawn, peripheral blood mononuclear cells (PBMCs), plasma heparin, plasma EDTA, and buffy coat will be isolated, according to standard procedures, and stored at the Laboratory of Biomarkers in a double-locked −80°C freezer at the Behavioral and Biomedical Sciences Building (BBSB) in the Faillace Department of Psychiatry at the UTHealth for future studies on biomarkers related to bipolar disorder. In addition to blood sample, all subjects and parents will be asked to provide a saliva sample, ~2 ml. Saliva samples will be collected only at baseline, using an Oragene DISCOVER (OGR-500/600) kit and cup, and stored at room temperature in a locked cabinet at the BBSB Wet Lab. They will remain at room temperature until processed for DNA. Once the samples are processed, they will be stored at the Laboratory of Biomarkers in a double-locked −80°C freezer until analysis. When the 20-year period ends, all biological samples will be destroyed.

##### Neuroimaging

The participants will complete a brain imaging scan, consisting of structural MRI, magnetic resonance spectroscopy (MRS), diffusion tension imaging (DTI), and resting-state functional MRI (fMRI). They will undergo the MRIs on a separate date, after completing the two initial baseline visits. Moreover, the subjects will also be invited to repeat the MRI portion of the study at their annual follow up visits. Neuroimaging studies will help in the investigation of volumetric and connectivity singularities associated with the diagnosis of BD, vulnerability for the disease, and brain imaging trajectories related to BD.

Table 1 provides a summary of the assessments, Table 2 describes the clinical evaluation instruments for the screening, second, and follow-up visits, and Table 3 provides the imaging parameters.

**Table 2:** The greater Houston Area Bipolar Registry – demographics, family history of psychiatric disorders and clinical evaluation instruments according to the assessment.

**Screening visit**		**Second visit**		**Annual Follow-up^b^**		
**Children/adolescents**	**Parents**	**Children/adolescents**	**Parents**	**Enrolled patients (6–17 years old)**	**Year follow up (18 years old or more)^c^**	**Parents^d^**
Demographics form, clinical history, developmental milestones	MINI International Neuropsychiatric Interview for DSM-5	Screen for Child Anxiety-Related Disorders-child version (SCARED)	Behavioral avoidance/inhibition scale (BIS/BAS)	Demographics form and clinical history update	Demographics form and clinical history update	MINI International Neuropsychiatric Interview for DSM-5
Family History of Psychiatric Disorders	Young Mania Rating Scale Version (YMRS)	Childhood Trauma Questionnaire (CTQ)	Disruptive Behavior Rating Scale (DBDRS)	Family History of Psychiatric Disorders	MINI International Neuropsychiatric Interview for DSM-5	Young Mania Rating Scale Version (YMRS)
MINI International Neuropsychiatric Interview for children and adolescents English version 7.0.2 for DSM-5 (M.I.N.I. KID)	Montgomery-Asberg Depression Scale (MADRS)	Stressful Life Events Schedule (SLES)	Screen for Child Anxiety-Related Disorders-parent version (SCARED)	MINI International Neuropsychiatric Interview for children and adolescents English version 7.0.2 for DSM-5 (M.I.N.I. KID)	Structured Clinical Interview for DSM-IV Axis II disorders SCID-II IV	Montgomery-Asberg Depression Scale (MADRS)
Clinical Global Impression-Severity	Clinical Global Impression (CGI)	Quick Inventory of Depressive Symptomatology (QIDS-SR)	Affective Reactivity Index (ARI)	Clinical Global Impression-Severity	Montgomery-Asberg Depression Scale (MADRS)	Clinical Global Impression (CGI)
Young Mania Rating Scale Version (YMRS)		Prodromal Questionnaire-Brief Child Version (PQ-B)	Peterson Pubertal Development Scale	Young Mania Rating Scale Version (YMRS)	Clinical Global Impression-Severity	Behavioral avoidance/inhibition scale (BIS/BAS)
Children's Depression Rating Scale, Revised (CDRS-R)		The Cambridge Neuropsychological Test Automated Battery-CANTAB^a^	Family Environment Scale	Children's Depression Rating Scale, Revised (CDRS-R)	Columbia Suicide Severity Rating Scale (C-SSRS)	Disruptive Behavior Rating Scale (DBDRS)
Columbia Suicide Severity Rating Scale (C-SSRS)			Stressful Life Events Schedule-parent version	Columbia Suicide Severity Rating Scale (C-SSRS)	WHOQOL-BREF	Screen for Child Anxiety-Related Disorders-parent version (SCARED)
Hollingshead Socioeconomic Status form			Self-Identified Stage of Recovery (SISR)	Hollingshead Socioeconomic Status form	Young Mania Rating Scale Version (YMRS)	Affective Reactivity Index (ARI)
Children's Global Assessment Scale			Attitudes Toward Seeking Professional Psychological Help Scale-Short form (ATSPPH-SF)	Children's Global Assessment Scale	Stressful life events scale (SLES)	Peterson Pubertal Development Scale
Pubertal Development Scale (Peterson Interview Version)			Child's Sleep Habits Questionnaire	Pubertal Development Scale (Peterson Interview Version)	Functioning Assessment Short Test (FAST)	Family Environment Scale
Modified Overt Aggression Scale (MOAS)				Modified Overt Aggression Scale (MOAS)	Barratt Impulsiveness Scale (BIS-II)	Stressful Life Events Schedule-parent version
Morisky Medication Adherence Scale (MMAS-8)				Morisky Medication Adherence Scale (MMAS-8)	Addiction Severity Index (ASI)	Self-Identified Stage of Recovery(SISR)
Quick Inventory of Depressive Symptomatology (QIDS-SR)				Screen for Child Anxiety-Related Disorders-child version (SCARED)	MOODS Self Report (MOODS-SR Lifetime)	Attitudes Toward Seeking Professional Psychological Help Scale-Short form(ATSPPH-SF)
Screen for Child Anxiety-Related Disorders-Child version (SCARED)				Childhood Trauma Questionnaire (CTQ)	Internal State Scale (v.2) (ISS)	Child's Sleep Habits Questionnaire
Stressful Life Events Schedule (SLES)				Stressful Life Events Schedule (SLES)	Morisky Medication Adherence Scale (MMAS-8)	
				Quick Inventory of Depressive Symptomatology (QIDS-SR)	Self-Identified Stage of Recovery (SISR)	
				Prodromal Questionnaire-Brief Child Version (PQ-B)	Attitudes Toward Seeking Professional Psychological Help Scale-Short form (ATSPPH-SF)	

a *Every 2 years*.

b *Will also be collected vital signs*.

c *For those 18 years or older will be applied The M.I.N.I. for DSM-5 instead of MINI International Neuropsychiatric Interview for children and adolescents English version 7.0.2 for DSM-5 (M.I.N.I. KID)*.

d *Parents will not complete a new M.I.N.I. for DSM-5 and will no longer be requested to complete self-reports once participants become 18 years old*.

**Table 3:** Imaging parameters.

**Sequence**	**TE (ms)**	**TR (ms)**	**Voxel size (mm)**	**Encoding b-factor (smm^**–2**^)**	**Matrix (x, y, z)**	**Scan time**
3D Sag T_1_-W	3.68	8.1	1 × 1 × 1	–	256 × 256 × 180	251
3D Sag Flair	292.5	4,800	1 × 1 × 1	–	256 × 256 × 180	317
Axial 2D-Turb-SE	20/90	6,800	1 × 1 × 2	–	256 × 256 × 80	231
DWI	65	9,000	1 × 1 × 2	Icosa21bpn_ord (42 directions)	256 × 256 × 80	528

### Statistical Analysis

Analyses will primarily rely on generalized linear mixed modeling (GLMM) to evaluate the probability of observing each diagnosis over time as a function of different predictors (clinical; cognitive; blood and saliva; neuroimaging). Model selection via fit indices will be used to specify the random effect structure of the model (e.g., level 2 intercepts and slopes). Models will establish the functional form of time, whereby polynomial or spline terms will be used to model non-linear effects. Observed group will then be modeled as a function of the interaction between the functional form of time and a given predictor, controlling for constituent lower-order main effects and potential confounding variables. If the interaction between time and predictor is supported, follow-up models will evaluate simple effects of time within different strata of the predictor. Otherwise, models will drop the interaction term to reduce to main effects only. The broadest analysis will use the multinomial distribution to fit the three-level outcome variable, with follow-up pairwise comparisons between groups modeled via the binomial distribution (i.e., logistic regression). Optimized multiple predictor models will be established via penalized regression techniques as described in the literature (35, 42).

Modeling assumptions will be evaluated via visual assessment of graphical plots and formal statistical tests. Violated assumptions will be addressed via variable transformation, stratification, and/or model respecification. Missing data will be handled via maximum likelihood, explicit modeling of missingness, and imputation where appropriate. False discovery rate will be used to adjust for Type I error across multiple comparisons within families of statistical tests. Potential confounding variables will be included as needed. Preliminary analyses will evaluate relationships between baseline characteristics, observed group, and outcomes. Any baseline characteristic that demonstrates a relationship with both the observed group variable and the outcome variable in a given model will meet criteria for confounding. In this case, models will be fit with and without covariate adjustment. If inferences are impacted due to adjustment, both models will be reported; otherwise, the simpler model without adjustment will be reported. Finally, theoretically intractable covariates will be included as a default where necessary (e.g., sex and age for neuroimaging outcomes).

### Power and Sample Size Considerations

Sample size considerations for the current protocol are calculated via *k* = 1,000 Monte Carlo simulations in R and focus on evaluating the potential for differential conversion to bipolar disorder between healthy controls and unaffected offspring due to the influence of a given z-scored predictor. This predictor is hypothesized to exert greater influence on conversion rates for unaffected offspring (relative to healthy controls). Given the prevalence of BD in the general population of 4.4% (43), an average conversion probability for UO of 24.5% (15), and a significance level α = 0.05 (two-tailed), an evenly allocated sample size *N* = 112 would be required to achieve ≥80% power to detect the differences in these conversion probabilities.

## Discussion

The understanding of the neurobiology and the identification of variables associated with different outcomes and disease trajectories in BD is essential to successfully address their associated high mortality and morbidity (44). As the disease usually has an early age of onset, studies with young bipolar participants, as well as high-risk populations, provide a valuable opportunity for the identification of clinical and biological markers, which might contribute to the prevention and early identification of BD. Studies integrating different clinical and neurobiological data, digital media habits, and information on environmental factors could help to provide more accurate information for prevention, early diagnosis, and individualized response to effective treatments (12, 45, 46). The Greater Houston Area Bipolar Registry intends to assist in this worldwide effort investigating the clinical course and neurobiological mechanisms of affected and high-risk children and adolescents for BD; some superb longitudinal studies that have already been conducted over the last decades, results from a solid effort from extraordinary researchers and institutions worldwide, and our initiative will increase our current understanding of the neurobiology and trajectories of BD by identifying new biomarkers encompassing different modalities, including clinical, cognitive, biological and neuroimaging markers, and validating biomarkers already identified by the previous discussed studies. For instance, the Bipolar Illness Onset (BIO) study is a cohort study that aims to identify biomarkers for diagnostic discrimination between individuals with BD and both high-risk (unaffected offspring) and low-risk controls, as well as between different states of the illness (manic, depressive and remitted). Patients are currently being recruited from the Copenhagen Affective Disorder Clinic in Denmark. The study investigators are following individuals with BD, first-degree relatives (siblings or offspring), and controls with no history of affective disorders for 5–10 years. With the use of peripheral blood makers, structural and functional neuroimaging, neurocognitive, and smartphone-based data, the study investigators will also search for biomarkers able to predict the onset of the disorder and also for biomarkers associated with neuroprogression (47). The Canadian High-Risk Offspring Cohort study has published several studies from observations from up to 21 years of follow-up of unaffected young offspring with parental history of bipolar disorder and of healthy controls, providing a substantial contribution to the field (15). For instance, the authors have reported a 24.5% cumulative incidence of BD spectrum among high-risk offspring, the median age of illness onset of BD at ~21 years old, and an almost 2-fold increase in the risk of BD in individuals with prior sleep and anxiety disorders. The results of the Canadian High-Risk Offspring Cohort reiterate the increased risk associated with parental history of psychiatric disorder, as previously reported among the offspring of patients with major depressive disorder (48).

Horwitz et al. (49) described the design and initial screening results of the Longitudinal Assessment of Manic Symptoms (LAMS) Study (49). Parents of more than 2,600 children (6–12 years old) in the United States were asked to evaluate whether their children screened positive for elevated symptoms of mania according to the Parent General Behavior Inventory 10-Item Mania Scale (PGBI-10M), a rating scale used for assessment of manic symptoms. Positive screened, and a matched sample of negative screened children, were invited to enroll in the follow-up study. The authors reported a high frequency of children with elevated symptoms of mania (43%) according to a psychometric scale, as well as items that best discriminate positive and negative screened children (49). More recently, results from a *consortium* including the LAMS and another study, the Bipolar Offspring Study (BIOS), identified differences in functional connectivity during emotion regulation tasks between offspring of parents with BD, compared with offspring of parents with non-BD psychiatric disorders and healthy controls (50). Longitudinal findings from the LAMS study also identified substantial impairments associated with early attention-deficit/hyperactivity disorder (ADHD) comorbid with BD, as well as an increased risk for substance use associated with family environment burden, sustained high manic symptoms, and comorbid disruptive behavior disorder (51, 52). Over the 12-year follow-up of the Dutch Bipolar Offspring Study, Mesman et al. (53) reported that 13% of the sample met criteria for a bipolar spectrum disorder, and more than 70% fulfilled criteria for an Axis I psychiatric disorder. About one quarter had a lifetime diagnosis of substance use disorder, with a mean age of hypomania and mania onset of ~17 and 20 years old, respectively (53). The Dutch Bipolar Offspring Study had the advantage of following high-risk children and adolescents (offspring from parents with BD I or II) into adulthood (mean age of 28 years old) (53). Other findings from this study include depressive symptoms as a predictor of BD conversion and the association between life events and risk for first mood episode (54, 55). Birmaher et al. (56) assessed the lifetime prevalence of psychiatric disorders in offspring of parents with BD. From the children and adolescents 6–18 years old evaluated, the authors found a higher likelihood of bipolar spectrum disorders and Axis I psychiatric disorder, as assessed by the Structured Clinical Interview-DSM-IV (SCID) (57) among high-risk offspring when compared to offspring of control parents (56). This longitudinal study also showed lower cohesion and higher conflict in families of parents with psychiatric disorders when compared to healthy controls, as well as a greater number of severe stressful life events in offspring of parents with BD (58, 59). In addition to allowing the replication of findings already reported and to test new hypotheses with a diversity of clinical and biological data, the marked ethnic diversity that enriches the greater Houston area allows studying a population that reflects a world that has become, progressively, more globalized. We are committed to this endeavor with a diverse and highly technical research team, willing to work in collaboration with other research groups.

## Ethics Statement

An ethics committee approved the study under the number HSC-MS-12-0673. This updated version follows the tenants of the Declaration of Helsinki and is in the process of submission to the UT Health Institutional Review Board. We expect to follow the participants of the study for up to 20 years, depending on the availability of funds. This timeline covers the age in which the first peak of onset of the disease (early adulthood – 15–24 years old) is more common (60).

## Author Contributions

AD drafted a first version of the manuscript. RS, GZ-S, IK, JQ, MS, and JS conceived of and designed the protocol. AD, VC, KD, EV, BF, and GS provided feedback and additions to the protocol and the text. All authors contributed to the article and approved the submitted version.

## Conflict of Interest

The authors declare that the research was conducted in the absence of any commercial or financial relationships that could be construed as a potential conflict of interest.
